# Capture of Essential Trace Elements and Phosphate Accumulation as a Basis for the Antimicrobial Activity of a New Ultramicrobacterium—*Microbacterium lacticum* Str. F2E

**DOI:** 10.3390/microorganisms10010128

**Published:** 2022-01-08

**Authors:** Nataliya E. Suzina, Andrey V. Machulin, Vladimir V. Sorokin, Valentina N. Polivtseva, Tatiana Z. Esikova, Anna P. Shorokhova, Yanina A. Delegan, Tatiana N. Abashina

**Affiliations:** 1 Federal Research Center “Pushchino Scientific Center for Biological Research of the Russian Academy of Sciences”, Skryabin Institute of Biochemistry and Physiology of Microorganisms, Pushchino 142290, Russia; and.machul@gmail.com (A.V.M.); kaistia@gmail.com (V.N.P.); das3534@rambler.ru (T.Z.E.); shorann@rambler.ru (A.P.S.); mewgia@yandex.ru (Y.A.D.); tnabashina@gmail.com (T.N.A.); 2 Federal Research Center of Biotechnology of the Russian Academy of Sciences, Winogradsky Institute of Microbiology, Moscow 117312, Russia; vlvlsorokin@gmail.com

**Keywords:** intermicrobial interaction, antagonistic effects, ultramicrobacteria, ultrastructural organization

## Abstract

Microbial interactions play an important role in natural habitat. The long-term coevolution of various species leads to the adaptation of certain types of microorganisms as well as to the formation of a wide variety of interactions such as competitive, antagonistic, pathogenic and parasitic relationships. The aim of this work is a comprehensive study of a new ultramicrobacterium *Microbacterium lacticum* str. F2E, isolated from perennial oil sludge, which is characterized by high antimicrobial activity and a unique ultrastructural organization of the cell envelope, which includes globular surface ultrastructures with a high negative charge. A previously undescribed mechanism for the antagonistic action of the F2E strain against the prey bacterium is proposed. This mechanism is based on the ability to preferentially capture essential microelements, in which charge interactions and the property of phosphate accumulation may play a significant role. The revealed type of intermicrobial interaction can probably be attributed to the non-contact type antagonistic action in the absence of any diffuse factor secreted by the antagonistic bacteria.

## 1. Introduction

A comprehensive study of various aspects of intermicrobial antagonistic interactions in natural habitats and the fundamental principles underlying them is an actual task which provides new insights into the principles of microbial ecosystems functioning. The results obtained can be used in plant protection measures, as well as in medicine and veterinary to develop approaches using new biologically active compounds or their complexes for the treatment of dangerous infectious diseases [1]. Research in this direction is especially relevant in connection with the emerging problem of a large-scale increase in the resistance of human bacterial pathogens to known antibiotics. In addition, the data obtained can be used for the effective destruction of biofilms of pathogenic forms of bacteria in humans and animals, which are not available in this form for exposure to antibiotics. In such cases some new isolates of predatory forms of bacteria can be utilized as “live antibiotics” [1]. In a specific natural biotope, microorganisms are rarely found in the form of a homogeneous population [2]. Therefore, there is a wide variety of types of microbial interactions, both with higher organisms and at the interspecies microbial level, which contain a lot of mechanisms (including molecular ones), many of which are described and systematized [2,3].

Among the huge number of various types of antagonistic intermicrobial (as well as interorganismic and intercellular) interactions, interbacterial interactions of the predator-prey type are of particular interest. Predatory bacteria are phylogenetically very diverse and widespread in terrestrial and aquatic environments [4,5,6,7,8]. In addition, predatory bacteria have been found in extreme biotopes, for example, under anaerobic conditions [9,10,11]. To date, it is known that bacterial predation can be clearly distinguished into obligate and facultative. In addition, it was proposed to divide bacterial predators into four main types according to their feeding strategy [12]: “wolf pack”, epibiotic, direct invasion, and periplasmic [13]. However, it is difficult to draw clear boundaries delineated these strategies. Undoubtedly, most researchers in each case suggest the existence of many variations of strategy types. In addition, fundamentally new previously undescribed strategies are also possible.

The authors of work [14] suggested that predatory bacteria can exhibit very different phenotypes and surprising physiological adaptations. According to [15,16], predation as well as various including still unknown antagonistic intermicrobial interactions are a common feature of prokaryotes; such bacteria can be found in a large number of phylogenetic groups.

To discover the bacterial predators with unknown antagonistic strategy, a complex of modern microscopic methods, including light and transmission electron microscopy, original microbiological approaches and molecular biology methods are used. Here, we describe a new mechanism of antibacterial action, which is based on a non-contact antagonistic action in the absence of any secreted diffusible factor.

## 2. Materials and Methods

### 2.1. Strain Isolation and Culture Conditions

To isolate the strain F2E, oil sludge (sludge enriched with oil products) was used. Strain F2E has been stored in the field conditions for more than 35 years (Nizhnekamsk, Tatarstan, Russia) and contains up to 40% of organic substances, about 30% of which, in turn, make up petroleum products. To obtain an enrichment culture, 10 g of the sample was added to a IBPM RAN (5/5) tryptone–soybean medium (containing 30 g/L of soybean extract, 5 g/L of casein hydrolysate, 1 g/L of yeast extract, and 60 mL/L of amino peptide (pH 8.0)) diluted 5–10 times and cultured in Erlenmeyer flasks with 200 mL of medium for 10–14 days at a temperature of 24 °C with stirring at 180 rpm. Further, the enrichment culture was spread to individual colonies on an agar medium of the same composition. Bacterial cells from the grown colonies were observed under a phase contrast microscope, and ultra-small bacteria were selected. Then a pure culture of the isolate selected in this way was obtained, which we designated as the strain F2E.

The strain F2E was cultured on the 5/5 agar medium and BBL Nutrient Agar (Becton Dickinson Microbiology System, Franklin Lakes, NJ, USA).

### 2.2. Phylogenetic Analysis

Genomic DNA was isolated from cells using the Fungal/Bacterial DNA Kit (Zymo Research, Irvine, CA, USA) according to the manufacturer’s recommendation. The 16S rRNA gene was amplified by PCR using primers universal for 16S rRNA prokaryotes: 27f (5′-AGAGTTTGATCCTGGCTCAG-3′) and 1492r (5′-TACGGYTACCTTGTTACGACTT-3′) [17]. The amplified DNA was purified using the Zymoclean Gel DNA Recovery Kit (Zymo Research, Irvine, CA, USA). Sequencing of PCR DNA fragments was performed on an Applied Biosystems 3130 Genetic Analyzer automatic sequencer.

Primary phylogenetic screening of the obtained sequences was performed using the BLAST program (http://www.ncbi.nlm.nih.gov/blast, accessed on 1 January 2022) and the EzBioCloud database (www.ezbiocloud.net, accessed on 1 January 2022). For phylogenetic analysis, 16S rRNA gene sequences were taken from the GenBank database (www.ncbi.nlm.nih.gov, accessed on 1 January 2022). The nucleotide sequences of the 16S rRNA gene obtained for the F2E strain were manually aligned with the sequences of reference strains of the related microorganisms. Phylogenetic tree constructed using partial 16S rRNA gene sequences by the neighbor-joining method with a bootstrap test of 1000 replicates was performed using MEGA 10.0 (https://www.megasoftware.net, accessed on 1 January 2022).

The 16S rRNA gene of *Microbacterium* sp. F2E has been deposited in the NCBI GenBank database under the number OL454499.

### 2.3. Microscopy

#### 2.3.1. Phase Contrast Microscopy

Microscopic studies were conducted using a Nikon Eclipse Ci microscope (Nikon, Tokyo, Japan) with a ProgRes SpeedXT camera (Jenoptic, Jena, Germany).

#### 2.3.2. Transmission Electron Microscopy

In the intact state, the cells of the isolate were studied in the form of unstained total preparations and in the form of negatively stained preparations. Negative staining was performed with 0.2% uranyl acetate aqueous solution. To prepare ultrathin sections, the cells of the tested variants including cyst-like cells were concentrated by centrifugation (10,000× *g*, 15 min) and fixed in 2% glutaraldehyde solution in 0.05 M cacodylate buffer (рН 7.2) for 1 h at 4 °C. Thereupon, the samples were washed three times with 0.05 M cacodylate buffer (рН 7.2) and additionally fixed with a 2% OsO_4_ solution in the same buffer for 4 h at 18–20 °C. Glutaraldehyde–osmium fixation in the presence of ruthenium red was used for contrasting cell wall polysaccharides and the S-layer [18]. Dehydrated samples in agar blocks were subsequently encapsulated in Epon 812 epoxy resin. The sections were mounted on support grids, contrasted for 30 min in 3% uranyl acetate solution in 70% alcohol, and additionally contrasted with lead citrate according to Reynolds [19]. Ultrathin sections and negatively stained preparations were examined under a JEM-1400 transmission electron microscope (JEOL, Tokyo, Japan) at an accelerating voltage of 80 kV.

#### 2.3.3. X-ray Microanalysis

X-ray microanalysis of the elemental composition of acidocalcisome-like ultrastructures on thin sections was carried out without additional contrasting using a JEM-1400 microscope (JEOL, Tokyo, Japan) equipped with an X-ray microanalyzer (Oxford Instruments, Abingdon, UK) at an accelerating voltage of 80 keV.

### 2.4. Cell Volume Calculation

Cell volume calculation was based on the data of morphometric analysis of the electron microscopic images of negatively stained or total bacterial preparations. While calculating cell volume (*V*), it was assumed that they represented spheres (1) or rod-shaped (2). The following formula was used:

(1) $$V=\frac{1}{6}\pi d^{3}$$

(2) $$V=\frac{1}{6}\pi ld^{2}$$

where *l* is the cell length, and *d* is the cell diameter.

### 2.5. Physiology and Biochemical Assay

The liquid 5/5 medium was used for determining optimum growth conditions for the tested strain. The temperature optimum was established by varying the temperature within the 4–42 °C range, and the effect of pH on growth was assessed within the 4.0–11.0 range. The medium pH was changed by adding 3 M NaOH or 1 M HCl. Halotolerance was determined by cultivation in the media containing 0–15% NaCl. Growth intensity was assessed by measuring the optical density at 590 nm in a 5 mm cuvette on a UV Specord spectrophotometer (Carl Zeiss, Jena, Germany).

To determine the spectrum of utilized substrates and enzyme activities of the isolate, we used the API 20E and API 50CH tests (bioMerieux, Marcy-l’Étoile, France) in compliance with the manufacturer’s instructions.

The presence of catalase was qualitatively determined by monitoring bubble formation upon adding a drop of 3% *v*/*v* H_2_O_2_. Oxidase activity was determined by the technique based on the color reaction that is associated with the oxidation of tetramethyl-p-phenylenediamine. Hydrolysis of cellulose, casein, starch, and Twin 20, 40, 60, and 80 was performed as described by Gonzalez et al. [20].

### 2.6. Antibiotic Resistance Testing

To analyze the resistance of the strains F2E to antibiotics, 100 µL of the culture in the exponential growth stage (CFU 1.2 × 10^9^) was applied to Petri dishes with 5/5 solid medium and evenly distributed over the entire surface of the dish using the spatula. The antibiotic discs (CJSC NICF, St-Petersburg, Russia) were laid out on top of the culture at an equal distance from each other and from the edges of the Petri dish. The results were evaluated after 24 h of culture at 25 °C by the presence of a lysis zone around the disc. If such zone was absent, the strain had resistance to a given concentration of antibiotic.

### 2.7. Antagonistic Activity Assay

Antagonistic activity of the strain F2E was assessed by two methods. One of them it was the well diffusion method [21]. For this purpose, 10–15 µL of strain F2E culture was introduced into the wells, and, after diffusion of the F2E cells into the agar, it was covered with semisolid nutrient agar (0.7%) containing (5–7) × 10^7^ cells/mL of the test bacterial culture. Inoculated media were incubated at 24 °C overnight. The antagonistic activity of strain F2E was estimated from the presence of growth inhibition zones around the wells.

The second method estimated the antibacterial activity of the strain F2E based on the investigation of the formation of the test culture lysis zone. For this study, the strain F2E was preliminarily grown in a 5/5 liquid medium. Then, studies were carried out on the two ways: (1) suspension of the strain F2E in the exponential growth phase directly was taken for investigation of the antagonistic activity and (2) bacterial suspension was preliminary precipitated by centrifugation (10,000× *g*, 10 min), supernatant was pass through membrane filters (“Millipore”) with a pore diameter of 0.4 µm, and then the supernatant containing the products of the secondary metabolism was used in the experiment to determine the antibacterial activity. Then, on standard Petri dishes with a solid medium 5/5, 100 µL of the test culture in the exponential growth stage was applied and evenly distributed over the entire surface of the dish with the spatula. Sterile filter paper disks were laid on top of the test culture under study, and 5 µL of the bacterial suspension or supernatant was applied to them. The results were evaluated after 24 h of culture at 25 °C by the presence of a lysis zone around the paper disk. The presence of such zone means the activity of the strain F2E in relation to the test culture.

As test cultures, we used 22 strains of Gram-negative bacteria: *Alcaligenes faecalis* VKM B-1518, *Erwinia carotovora* B15, *E. herbicola* ATCC 27155, *Escherichia coli* K12, *Pseudomonas aeruginosa* PAO1, *P. alcaligenes* VKM-1295, *P. chlororaphis* PCL1391, *P. protegens* 38a, *P. putida* KT2422 and Gram-positive bacteria *Arthrobacter* sp. B52, *Bacillus cereus* GA5T, *B. megaterium* VKM B-512, *B. subtilis* ATCC 35646, *B. thuringensis* ATCC 35646, *B. weihenstephanensis* KBA4, *Lysinibacillus sphaericus* VKM В509, *Microbacterium liquefaciens* Ash10-2, *Micrococcus luteus* VKM B-1891, *M. roseus* VKM B-1236, *Rhodococcus erythropolis* Sh5, *Staphylococcus aureus* 209-P, and *Streptococcus salivarius* M15 received from different national collections of microorganisms and from the working collection of the authors (Table S1). The strains were selected according to their belonging to both Gram-positive and Gram-negative bacteria, as well as belonging to conditionally pathogenic microorganisms. The antibacterial activity was tested eight times to check the possible loss of antimicrobial activity by the cells of the F2E strain.

### 2.8. Determination of Metal Content

Strain F2E was preliminarily grown on agar medium 5/5 at 28 °C for 24 h. The biomass of the strain F2E was collected, then it was resuspended in sterile tap water and divided into 5 tubes, 10 mL each. In the first tube, 0.5 mL of 1% solution (copper sulfate) CuSO_4_ was added. In the second tube 0.5 mL of 1% (nickel chloride) NiCl_2_ solution (the final concentration of NiCl_2_ in the test tube was 0.005 g) was added. In the third tube, 0.5 mL of iron sulfate solution at a concentration of 0.375 g/5 mL and 200 μL of 10% H_2_SO_4_ solution were added. To the fourth tube 1 mL of a chelated iron solution (FeEDTA) at a concentration of 0.6 g/10 mL was added. The fifth test tube was a control containing only the biomass of the strain F2E suspended in the water.

A metal content in the samples was measured spectrophotometrically immediately after the preparation of the reaction mixture and after 1 h of incubation on a Shimadzu 1800 spectrophotometer (Shimadzu, Kyoto, Japan) in a 1 cm wide cuvette.

Nickel was measured according to the method from the work of Reznik [22]. The measurement mixture contained 50 µL of sample, 0.5 mL of 5% NaOH solution, 0.5 mL of 5% (NH_4_)_2_S_2_O_8_ solution, 0.5 mL of 2% dimethylglyoxime solution in 5% NaOH and 3.4 mL of water. The measurements were carried out at a wavelength of 460 nm.

The copper content was measured according to the method from the work [23]. The measurement mixture contained 100 mL of sample, 1 mL of 10% NH_3_ solution and 3.9 mL of water. Measurements were carried out at a wavelength of 590 nm.

The iron content was measured according to GOST 13195-73 [24]. The measurement mixture contained 100 mL of sample, 0.5 mL of 10% HCl solution, 5 mL of H_2_O_2_, 0.4 mL of 1% solution of potassium ferrocyanide trihydrate (C_6_H_6_FeK_4_N_6_O_3_) 1% and 9 mL of water, incubation was carried out for 30 min. Measurements were carried out at a wavelength of 600 nm.

### 2.9. DNA Sequencing, Assembly and Annotation of Complete Genomes

Genomic DNA of the strain F2E was isolated from the biomass of a fresh culture grown from one colony using the QIAamp DNA Mini Kit (cat. #51304; Qiagen, Hilden, Germany). The libraries were prepared using the Nextera DNA Library Preparation Kit. The concentration of the libraries was determined using a Quantus fluorometer, the quality of the libraries was assessed using QIAxcel gel electrophoresis. Sequencing was performed on Illumina HiSeq500 equipment at the BioSpark biotechnological laboratory (Troitsk, Moscow, Russia). The processing and assembly of genomes was carried out at the Laboratory of Physiology of Microorganisms of the Institute of Biophysics and Microorganisms, Russian Academy of Sciences.

Quality control of readings was performed using FastQC software (http://www.bioinformatics.babraham.ac.uk/projects/fastqc, accessed on 1 January 2022). Duplicate reads removed with Clumpify from BBmap package (https://sourceforge.net/projects/bbmap/, accessed on 1 January 2022). Low quality (Q < 10), short (<50 bp) reads and adapter sequences were removed using the Trimmomatic software [25]. The number of reads before and after quality control (QC) is 25 786 039 and 22 365 895 respectively, length of readings was 100 bp.

Raw filtered reads of the strain F2E were collected using SPAdes version 3.15.2 software [26] at a k-measure size of 95 (Table 1).

To assess the completeness of using raw readings in an assembly, raw filtered readings of the strain F2E were mapped to the corresponding assembly using the Bowtie2 ver. 5.1 [27]. Minor assembly errors were fixed using the Pilon 1.23 software [28].

Transport and ribosomal RNAs of the strain F2E were identified using tRNAscan-SE [29] and RNAmmer v1.2 (https://services.healthtech.dtu.dk/service.php?RNAmmer-1.2, accessed on 1 January 2022), respectively. The assembled genome was annotated using Prokka [30] and RAST [31]. The phylogenetic tree of the complete genome was built using the Realphy web service [32]. The genome sequences of related strains required for constructing a phylogenetic tree were taken from the Genbank database.

Average nucleotide identity (ANI) value was calculated using the EzBioCloud web service [33]. The value of digital DNA-DNA hybridization (DDH) was calculated using the Genome-to-Genome Distance Calculator 2.1 [34]. The Kyoto Encyclopedia of Genes and Genomes (KEGG) [35] was used for functional annotation.

The complete genome of *Microbacterium* sp. F2E has been deposited in the NCBI GenBank database under the number JAJJIB000000000.

The genome sequences of *M. lacticum* DSM 20427 and *Microbacterium* sp. F2E were automatically annotated using NCBI prokaryotic genome annotations (https://www.ncbi.nlm.nih.gov/genome/browse#!/prokaryotes/, accessed on 1 January 2022).

### 2.10. Genome Comparative Analysis

For the whole-genome comparative study, genomes of *M. lacticum* DSM 20427 was retrieved from the NCBI database (NZ_VFPS00000000.1) and compared with the genome of the study *Microbacterium* sp. F2E (GenBank: JAJJIB000000000.1). Homology searches and searches of the unique genes were performed with BLAST (https://blast.ncbi.nlm.nih.gov/Blast.cgi, accessed on 1 January 2022) and MEGAX software (https://www.megasoftware.net/, accessed on 1 January 2022) using the default settings.

## 3. Results

### 3.1. Morphology and Ultrastructural Organization

The strain F2E was isolated from a sample of oil sludge aged 35 years (Nizhnekamsk, Tatarstan, Russia). The cells of the strain F2E are represented mainly by short, irregularly dividing rods. Division occurs by the formation of a septum with the divergence of the divided cells at an angle to each other and the formation of daughter cells of a spherical shape or, more often, in the form of short rods. The size of daughter cells is 0.3–0.45 × 0.28–0.3 μm. The cell volumes calculated on the basis of their linear dimensions according to the formulas for the volumes of rod-shaped and spherical cells (see the Methods section) are 0.080 ± 0.007 μm^3^, which makes it possible to classify this bacterium as ultramicrobacterium, since their cell volumes are significantly less than 0.1 μm^3^ under conditions growth on media of different composition and concentration [16].

**Figure 1:**
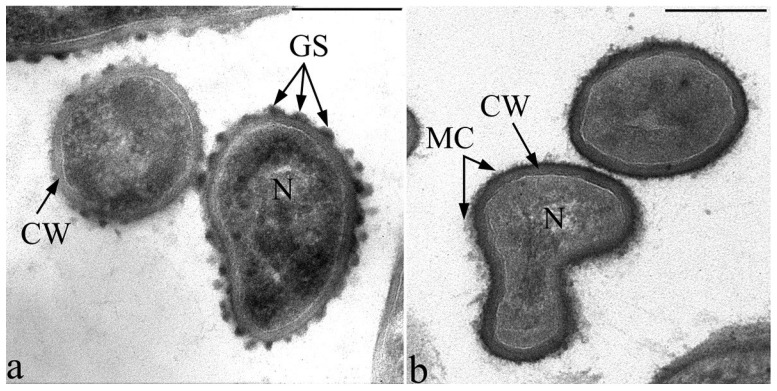
Transmission electron microscopy. Ultrathin sections of the strain F2E cells (**a**) and cells of the most phylogenetically close type strain of *M. lacticum* VKM Ac-1145 (**b**), fixed in the presence of ruthenium red. Electron-dense globular structures (GS) on the surface of the cell wall of strain F2E (**a**) are indicated. Designations: GS—globular structure; CW—cell wall, MC—microcapsule, N—nucleoid. Bar—200 nm.

The cell wall of the strain F2E has a typical structure of Gram-positive bacteria. According to the electron microscopic analysis of ultrathin sections, the thickness of the bacterial cell wall is 30–32 nm (Figure 1a). Pili and flagella are absent. The fixation of bacteria in the presence of ruthenium red made it possible to identify electron-dense globules localized on the outer surface of the cell wall, which are closely associated with the cell wall, in fact, are included in its composition. These globular surface structures are characterized by an irregular, ovoid, slightly flattened shape. On ultrathin sections of cells of the type strain *M. lacticum* VKM Ac-1145, most phylogenetically close to the strain F2E, such superficial ultrastructures were absent (Figure 1b).

### 3.2. Identification, Physiology and Biochemical Properties of the Strain F2E

To determine the closest related strains, which were later used to construct a phylogenetic tree and calculate ANI and DDH, we performed a BLAST search for the 16S rRNA sequence of the strain F2E. For further analysis, we used type strains of related species, as well as strains with complete genomes.

To identify bacterial strains, we used an integrated approach, including: (1) comparison of the chromosomes of the studied strain and the closest related strains, (2) calculation of the ANI parameter values (average nucleotide identity), and (3) calculation of the DDH (digital hybridization) parameter values (Table 2).

**Figure 2:**
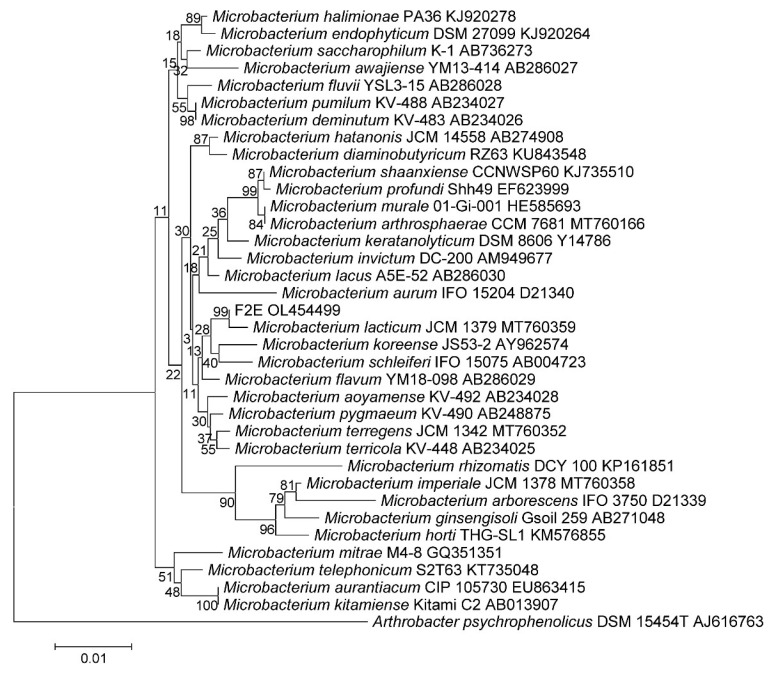
Neighbor-joining phylogenetic tree based on the 16S rRNA gene sequences showing the relationships of the strain F2E among members of the genus *Microbacterium*. Bar, 0.01 substitutions per nucleotide position.

According to the analysis of the nucleotide sequence of the 16S rRNA gene and the whole genome nucleotide sequence, the bacteria of the strain F2E are representatives of the genus *Microbacterium*, the closest phylogenetic related species is *M. lacticum* (99.9% similarity) (Figure 2).

The strain F2E is chemoorganotroph, aerobic, oxidase- and catalase-positive and urease-negative. It metabolizes a limited range of organic compounds. The strain F2E is able to use simple carbohydrates (maltose, lactose, some amino acids) as a carbon source and ammonium, nitrate nitrogen and some amino acids (leucine, threonine, alanine, isoleucine) as a nitrogen source. The strain F2E hydrolyses starch, but not gelatin, casein and Tweens (20, 40, 60 and 80). According to API 50CH test, the strain F2E is able to utilize d-glucose, d-mannose, d-sorbose, esculin, d-sucrose, d-trehalose, d-melecytosis, d-turanose. It has the tryptophanedaminase. Major cellular fatty acids are C_14–18_, prevailing C_19:0_ cyclo.

The maximum growth of cells of the strain F2E was observed in the media 5/5 and BBL Nutrient Agar, and the growth rate decreased on analogs of these media diluted 10 times. The strain F2E grew in media with NaCl concentration up to 1%; no growth was observed at higher concentrations. Growth occurs at 18–40 °C with optimum growth at 28–30 °C. The pH range for growth was 5.0–8.0 and the optimum pH for growth was 7.0.

The strain F2E is sensitive to a wide range of antibiotics (μg/mL): rifampicin (25), ampicillin (50), gentamicin (10), penicillin (10), chloramphenicol (20), novobiocin (50), claforan (50), tetracycline (10), carbenicillin (25), kanamycin (25) and cefazolin (25). Resistant to antibiotics (μg/mL): nalidixic acid (50), tetracycline (10) and streptomycin (50).

### 3.3. Genomic Features of M. lacticum Strain DSM 20427 and Microbacterium sp. F2E

The genomic features of *M. lacticum* DSM 20427 and *Microbacterium* sp. F2E are summarized in Table 3.

The genome of *M. lacticum* DSM 20427 consists of 2.87 Mb and the G+C content is 70% (Table 3), similarly to the *Microbacterium* sp. F2E (Table 2). The *M. lacticum* DSM 20427 genome contains 3047 genes, three rRNAs, and 47 tRNAs (Table 3). Strains were identified as *M. lacticum strain DSM 20427* and *Microbacterium* sp. F2E 98% (BLAST).

### 3.4. Identification of Iron Transport and Phosphate Accumulation Gene Homologues

The uptake of iron siderophores in bacteria requires two steps. Firstly, to cross the outer membrane, it associates with a specific outer membrane transporter and is transported into the periplasm. Secondly, it binds to a cognate periplasmic binding protein and is actively transported across the inner membrane by an ATP-binding cassette (ABC) transporter.

In silico analysis of the annotated genome sequence of *Microbacterium* sp. F2E (JAJJIB010000003.1) revealed three genes (in positions 116905-117753, 117750-118826, 118823-119881) encoding iron chelate uptake ABC transporter family permease subunit proteins and iron-siderophore ABC transporter substrate-binding proteins. According to KEGG (https://www.genome.jp/kegg/, accessed on 1 January 2022) these genes (ID KEGG: GKZ92_05020) associated with metallic cation, iron-siderophore and vitamin B12 transporters and iron complex transporter functions. Features of iron-siderophore ABC transporter substrate-binding protein is defined by the Fe^3+^-siderophore binding domain FhuD. Such proteins have been shown to function as initial receptors in ABC transport of Fe^3+^-siderophores in many eubacterial species. Analysis of the annotated genome sequence of *M. lacticum* strain DSM 20427 revealed seven genes (in positions 2604-3578; 3610-4644; 157553-158527; 158559-159296; 796492-797523; 797520-798596; 798686-0799651) also coding iron chelate uptake ABC transporter family permease subunit proteins and iron-siderophore ABC transporter substrate-binding proteins.

Analysis of the annotated genome sequence of *Microbacterium* sp. F2E (JAJJIB010000003.1) revealed the *ppk2* gene (109082-109891, JAJJIB010000007.1) and a gene in position 277603-278466 (JAJJIB010000001.1) encoding proteins of the polyphosphate kinase family. The activity of the kinase family proteins is associated with the metabolism of polyphosphates. In addition, in the position 80038-81141 (JAJJIB010000001.1) gene *pstS* was found. This gene codes phosphate ABC transporter substrate-binding protein PstS, which is a substrate-binding component of the ABC-type transporter complex. According to gene ontology analysis service GO (http://geneontology.org/, accessed on 1 January 2022), the main function of this protein is phosphate ion binding (GO:0042301) and the key role in the biological process is defined as phosphate ion transmembrane transport (GO:0035435).

### 3.5. X-ray Microanalysis Investigation of the Globular Structures

**Figure 3:**
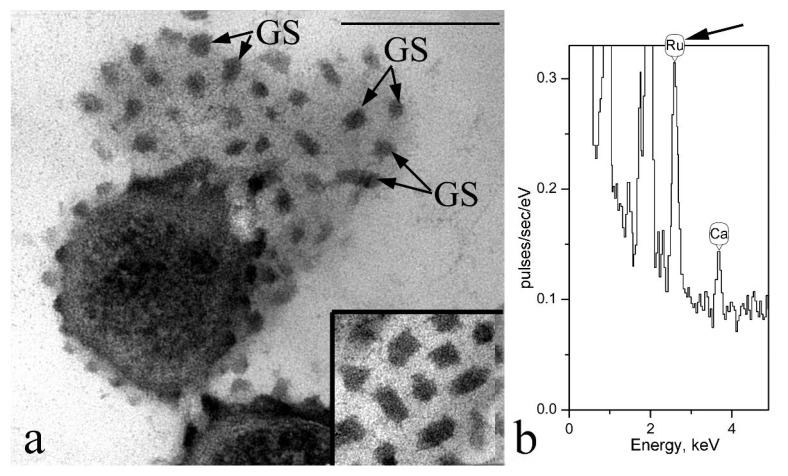
Ultrathin sections of cells of the strain F2E (**a**) Electron-dense globular structures (GS) on the surface of the cell wall of strain F2E are indicated. (**b**) X-ray spectrum obtained from electron-dense GS on a tangential (surface, oblique) slice (a slice fragment in the lower right corner of Figure 3a). The arrow indicates the peak of ruthenium. Designations: GS—globular structure. Bar—200 nm.

Our electron-cytochemical studies showed that ruthenium red selectively sits on globular structures (GS) localized on the cell wall surface of the strain F2E and does not bind to adjacent areas of the cell wall surface (Figure 1 and Figure 3). On sections tangential to the cell surface, it can be seen that the globules have angular bounding contours (Figure 3a). According to the analysis of ultrathin sections, the size of the globules can reach 40 nm in diameter (less often 50 nm). Globules were randomly distributed with a high density over the entire surface of the bacterial cell wall and were found at all stages of growth on nutrient media of different composition. It should be noted that during classical glutar-osmium fixation of cells of the strain F2E in the absence of ruthenium red, these structures were not detected. It is known that ruthenium red has a very high positive charge and is used in electron microscopy to study the localization of acidic (negatively charged) mucopolysaccharides in bacterial cells [36]. For this reason, we used the X-ray microanalysis method to investigate the possible “attraction” to GS and other positively charged ions such as Fe^2+^ and Cu^2+^, which, in particular, are essential for microorganisms.

In addition, investigations were carried out to study the possible preferential localization of nickel in the GS of the strain F2E during incubation in solutions containing nickel chloride, due to the possible high biotechnological potential of the strain F2E in measures to clean up the environment from toxic nickel-containing waste.

**Figure 4:**
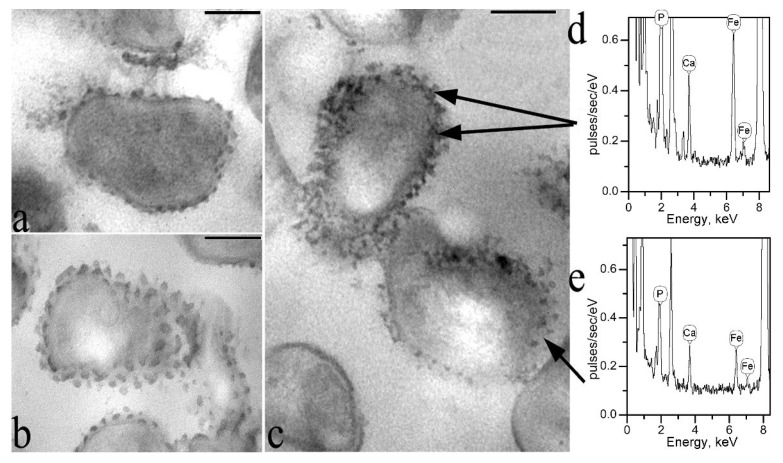
Transmission electron microscopy combined with X-ray microanalysis. Ultrathin unstained sections of the cells of the strain F2E after incubation in medium with copper sulfate CuSO_4_ (**a**); nickel chloride NiCl_2_ (**b**); with the mineral form of iron, ferrous sulfate FeSO_4_ (**c**); (**d**) X-ray spectrum of GSs in a medium with a mineral form of iron; (**e**) X-ray spectrum of the cell surface area between GSs in a medium with a mineral form of iron. Bar—200 nm.

Analysis of unstained ultrathin sections showed that in all investigated variants of incubation of the strain F2E in media containing compounds of copper, nickel, mineral and organic forms of iron, GS are clearly identified as electron-dense granules of characteristic shape and size. This suggests that all the studied elements bind to GS, as a result of which their electron density increases (Figure 4a–c). However, only in the case of incubation of the of the strain F2E cells in a solution with a mineral form of iron, the iron element was reliably detected by X-ray microanalysis in GS (Figure 4d,e). This is probably due to insufficient sensitivity of the device.

Studies by the method of X-ray microanalysis of total preparations of the cells of the strain F2E washed from the incubation medium, confirmed the binding of the studied elements to the cells of the strain F2E in each case (spectra are not shown). In the case of incubation in a CuSO_4_ solution, a change in the blue color of the solution was observed in the presence of cells of the strain F2E. In this case, the solution became discolored, and the cell suspension quickly self-precipitated and turned to a blue color.

**Figure 5:**
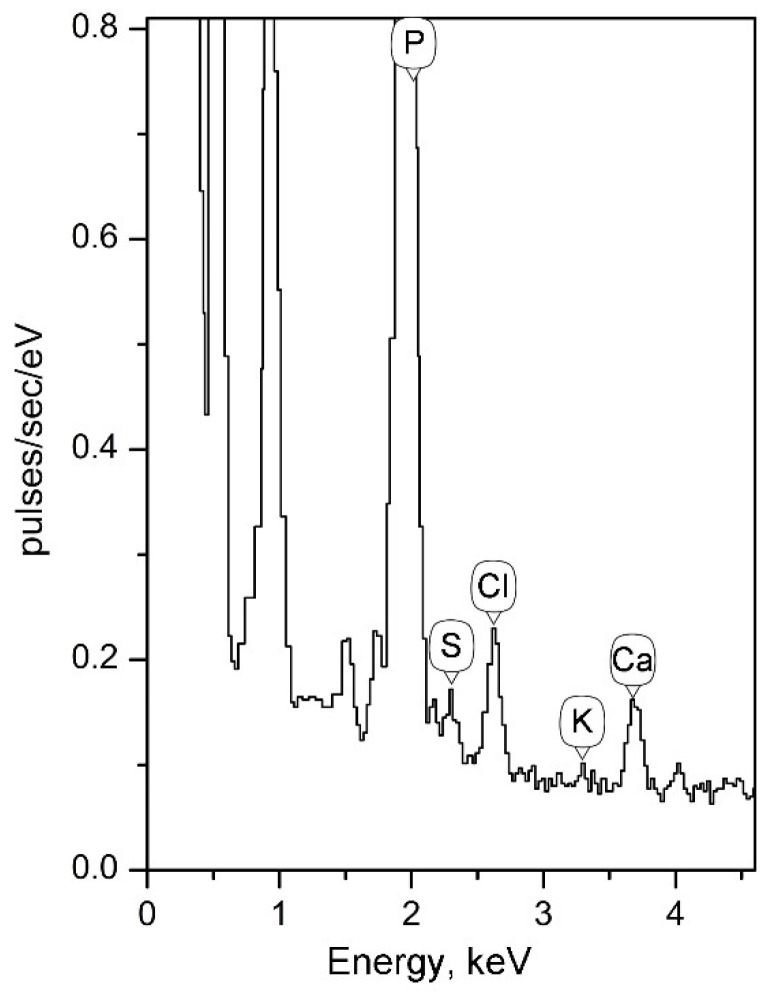
X-ray spectrum of a total cell preparation of the strain F2E.

In all experiments using the method of X-ray microanalysis, including sections and total preparations of studied cells, a very high peak of phosphorus was observed in the obtained spectra. As an example, a fragment of the X-ray spectrum of a total preparation of the strain F2E is shown (Figure 5). Cells in all studies were thoroughly washed from the components of the medium or incubation mixtures. These data indicate the ability of the strain F2E to accumulate large amounts of phosphorus from the environment. Phosphorus is an essential macronutrient. We cannot confirm the participation of GS in this process using the method of X-ray microanalysis, but it is clear that the strain F2E is an effective phosphate accumulator.

### 3.6. Antimicrobial Activity of the Strain F2E

**Figure 6:**
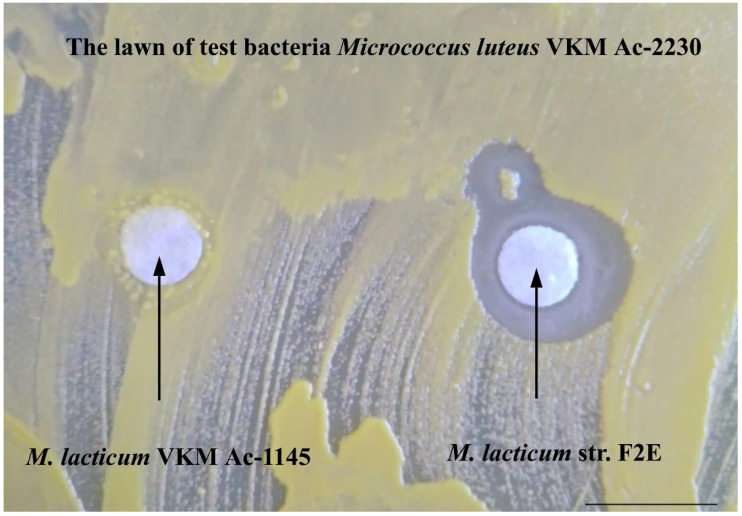
Inhibition of the growth of *M. luteus* VKM Ac-2230 by the strain F2E (24 h incubation, 28 °C). Bar—1 cm.

The strain F2E exhibited antimicrobial activity against all of the tested Gram-positive bacteria. The highest inhibited growth effect was identified related to *M. luteus* VKM Ас-2230 (Figure 6). Weak antimicrobial activity was revealed against some Gram-negative bacteria: *E. coli* K12 and two species of the genus *Pseudomonas* (*P. putida* KT2442 and *P. protegens* 38a) (Table 4).

Since the formation of the growth inhibition zones of test bacteria by the strain F2E could be caused by a number of reasons such as the action of synthesized extracellular lytic enzymes or low molecular weight peptide antibiotics secreted into the culture liquid, the antibacterial effect of the filtered culture liquid of the strain F2E was investigated.

Studies have shown that the culture liquid of the strain F2E without bacterial cells did not cause the formation of lysis zones or inhibition of the growth of test bacteria cells when growing on a solid medium. Thus, with a high degree of probability, it can be stated that the antagonistic effect of the strain F2E in relation to the test bacteria used in the work are not associated with the antibacterial effect of the components secreted into the culture liquid.

Experiments were also carried out with the identification of antibacterial activity against test bacteria in the typical strain of *M. lacticum* VKM Ac-1145, which is phylogenetically closest to the strain F2E, but does not have GS on the cell wall surface. In experiments with this strain, the formation of lysis zones or growth inhibition of test bacteria cells was not observed.

## 4. Discussion

Microbial interactions are an essential part of the ongoing processes in nature. The long-term coevolution of various species leads to the adaptation of individual types of microorganisms and to the formation of a wide variety of relationships, including competitive, antagonistic, pathogenic and parasitic relationships. Of particular interest is the role and mechanisms of ultramicrobacteria adaptation in natural biotopes. Ultra-small sizes (volumes) of cells provide certain ecological advantages: such microorganisms multiply more easily in natural conditions, occupying econiches that are inaccessible to other species. For ultramicrobacteria, an epibiosis-type predation mechanism has been described. This mechanism allows ultramicrobacterial cells to attach to the surface of large bacterial cell forms (“trapping nets”) and attack prey bacteria [7,37].

The new isolate, strain F2E, does not have an extensive polysaccharide network, flagella, fimbriae and pili, the cells of the strain are immobile. Long-term observations of the process of interaction between the F2E strain and the victim bacteria did not show contact between the cells. In addition, diffuse antimicrobial agent was not detected in the culture liquid of the strain F2E. The analysis of the carried out complex microbiological, physiological, biochemical, cytological and whole genome studies indicates a key role of GS in the mechanism of antagonistic action of the strain F2E cells.

To our knowledge, studies of such surface formations in other bacteria, similar to the globular ultrastructures of the F2E strain, have not been previously observed. However, specialized surface globular structures that play significant role in the metabolism of other bacterial species are described in the literature. These structures include “oxalosome”, localized on the outer layer of a bilayer cell wall of Gram-positive endospore-forming ammonium-dependent, obligately oxalotrophic bacteria of the genus *Ammoniphilus* [38], and cellulosome (the cellulose-binding, multicellulase-containing protein complex) in the bacterium *Clostridium thermocellum* [39]. They differ from the GS of the F2E strain in their organization, size, density and location on the surface. The most important difference between “oxalosome”, cellulosome and GS of the strain F2E lies in the detection way by TEM sections: oxalosome and cellulosome are detected on the cell surfaces using traditional glutar-osmium fixation. In coontrast, GS of the F2E strain were detected on the sections only in the presence of ruthenium red during cells preparation.

A comparative whole genome analysis of the studied strain *M. lacticum* str. F2E and the type strain of *M. lacticum* str. DSM 20427 showed in both cases the presence the system of siderophore genes associated with the capture and transfer of iron.

Data obtained indicate that siderophores do not play a key role in the mechanism of the antagonistic effect of the strain F2E, since for the type strain, in the presence of the same system of genes for the uptake and transfer of iron by siderophores, the formation of zones of inhibition or lysis was not observed under conditions of interaction with different studied victim bacteria.

The experimental data obtained demonstrate that the globular surface ultrastructures characteristic of the strain F2E are specialized nano-compartments of the cell wall with a high negative charge. This organization allows bacteria to preferentially capture essential microelements in their natural habitat, such as iron (Fe) and copper (Cu). In toxic (as a result of anthropogenic pollution) natural environments, accumulation of other positive ions on GS, such as nickel ions, can occur. The property to carry out mainly the accumulation of phosphorus is also apparently the reason for the occurrence of this macronutrient limit in the environment nearby to other types of cells. Our earlier studies of obtaining dormant forms of bacterial cells showed the effectiveness of the long-term incubation method in nutrient media limited in main vital macronutrients, such as nitrogen and phosphorus [40]. Under these conditions, non-spore-forming cells form cyst-like forms, obtain resistance to extreme environmental factors and ability to survive for a long time in unfavorable conditions [41]. The cells of *M. luteus* were the most sensitive to the nitrogen and phosphorus limits. Under the conditions of the limit for these two macronutrients, the population of *M. luteus* cells was almost entirely lysed, and only a few cells retained their viability and passed into a cyst-like resting state [42]. In our experiments on the interaction of the strain F2E and *M. luteus* VKM B1891 the lysis pattern was most clearly indicated (Figure 6), which also correlates with the property of the strain F2E to phosphate accumulation and the high sensitivity of micrococci to the phosphorus limit. It can be assumed that, in view of the different sensitivity of various types of microorganisms to the limitation of essential micro- and macro-elements, the other bacteria species cells in the immediate environment of the strain F2E can either lyse or pass into a dormant state.

## 5. Conclusions

On the example of a new ultramicrobacterium *Microbacterium lacticum* str. F2E isolated from perennial oil sludge, exhibiting antimicrobial activity against a wide range of bacteria, a new mechanism of intermicrobial antagonistic interaction based on fierce competition for essential micro- and macroelements is proposed. A distinctive feature of the antimicrobial activity of the F2E strain is the absence of direct contact with the cells of other bacteria and any diffuse factor secreted into the external environment with antimicrobial effect. Microbiological, physiological, biochemical and cytological studies indicate the key role of the globular structures (GS) in the mechanism of antagonistic action of cells of the F2E strain. The experimental data obtained demonstrate that the GS characteristics of the F2E strain are specialized nano-compartments of the cell wall with a high negative charge. The antagonistic effect of the F2E strain may be based on the ability to preferentially capture essential microelements by globular structures with a high negative charge and the ability of the cells of the F2E strain to accumulate phosphate.

## Figures and Tables

**Table 1:** Characteristics of draft assemblies of the complete genome of the F2E strain.

	F2E
Genome size, bp	2°873°295
Number of contigs	58
GC, %	70.4
The shortest contig, bp	502
Median sequence size, bp	11,735
Average sequence size, bp	49,539.6
Longest contig, bp	450,112
Contig N50	160,800
Contig L50	6

**Table 2:** Values of ANI and DDH parameters of the strain F2E and its related strains.

	ANI %	DDH %
*M. aurum* KACC 15219 (NZ_CP018762.1)	81.88	34.60
*M. aurum* DSM 8600T (JAFBCQ000000000.1)	82.18	34.50
*M. schleiferi* A32-1 (NZ_CP064760.1)	75.56	17.00
*M. oleivorans* A9 (CP031421.1)	77.23	22.20
*M. oleivorans* I46 (CP058316.1)	77.51	21.20
*M. oleivorans* NBRC 103075T (BCRG00000000.1)	77.27	22.30
*M. pygmaeum* DSM 23142T (NZ_LT629692.1)	76.07	19.40
*M. lacticum* JCM 1379T (BMOA00000000.1)	96.86	61.40
*M. hydrocarbonoxydans* DSM16089T (FNSQ00000000.1)	75.80	17.60
*M. caowuchunii* ST-M6T (NZ_CP044231.1)	76.12	19.00
*M. endophyticum* DSM 27099T (CP049255.1)	73.37	14.30

**Table 3:** Genomic features of *M. lacticum DSM 20427* and *Microbacterium* sp. F2E.

Genome Information	*Microbacterium* sp. F2E	*M. lacticum* DSM 20427
Chromosome size, Mb	2.87	3.09
Number of contigs	58	1
tRNAs	47	47
Noncoding RNAs	3	3
Complete rRNAs	3	3
Total genes	2795	3047
Total CDS	2742	2994
Coding CDS	2643	2813

**Table 4:** Antagonistic activity of the F2E strain.

Test Bacteria	Presence of Growth Inhibition Zone *
Gram-negative bacteria	
*Alcaligenes faecalis* E502	−
*Escherichia coli* K12	+/−
*Erwinia herbicola* ATCC 27155	−
*Erwinia carotovora* B15	−
*Pseudomonas putida* KT2442	+/−
*P. aeruginosa* PAO1	−
*P. alcaligenes* VKM-1295	−
*P. chlororaphis* PCL1391	−
*P. protegens* 38a	+/−
Gram-positive bacteria	
*Arthrobacter* sp. B52	+
*Bacillus megaterium* VKM B-512	+
*B. cereus* GA5T	+
*B. subtilis* ATCC 6633	+
*B. thuringiensis* ATCC 35646	+
*B. wehnestephanensis* KBAB4	+
*Lysinibacillus sphaericus* VKM B-509	+
*M. liquefaciens* Ash10-2	+
*Micrococcus luteus* VKM Ас-2230	+/+
*M. roseus* VKM B-1236	+
*Rhodococcus erythropolis* Sh5	+
*Staphylococcus aureus* 209-Р	+
*Streptococcus salivarius* M15	+

Note: * (−)—no growth inhibition zone; (+/−)—weakly inhibited growth zone (1–2 mm); (+)—growth inhibition zone (2–3 mm); (+/+)—highly inhibited growth zone (4–5 mm).

## Data Availability

The data presented in this study are available on request from the corresponding author.

## Supplementary Materials

The following are available online at https://www.mdpi.com/article/10.3390/microorganisms10010128/s1, Table S1. List of test bacteria for antagonistic activity.
