# Supplementary material for: Capture of Essential Trace Elements and Phosphate Accumulation as a Basis for the Antimicrobial Activity of a New Ultramicrobacterium—Microbacterium lacticum Str. F2E
Source: Microorganisms. 2022 Jan 8;10(1):128. doi: 10.3390/microorganisms10010128 (PMC8777731; doi:10.3390/microorganisms10010128)
Supplement: Supplementary file 1 [file microorganisms-10-00128-s001.zip › microorganisms-1528455-supplementary.pdf]

Table S1. List of test bacteria for antagonistic activity

Gram-negative bacteria	Gram-positive bacteria
Aeromonas veronii	*Arthrobacter* sp. B52
Alcaligenes faecalis VKM B1518	*Bacillus cereus* GAST
Erwinia carotovora B15	*Bacillus megaterium*
Erwinia herbicola ATCC27155	*Bacillus sphaericus* VKM B509-1
Escherichia coli K12	*Bacillus subtilis*
Pseudomonas aeruginosa ML 4262	*Bacillus thuringensis* ATCC35646
Pseudomonas chlororaphis PCL 1891	*Bacillus weihenstephanensis* KBA4
Pseudomonas protegens 38a	*Deinococcus radiodurans*
Pseudomonas putida KT2422	*Micrococcus luteus* VKM B1891
Rhodococcus erythropolis Sh5	*Micrococcus roseus* VKM B1236
Shewanella putrefaciens	*Staphylococcus* St 35
